# DNMT3B System Dysregulation Contributes to the Hypomethylated State in Ischaemic Human Hearts

**DOI:** 10.3390/biomedicines10040866

**Published:** 2022-04-07

**Authors:** Estefanía Tarazón, Lorena Pérez-Carrillo, Isaac Giménez-Escamilla, María García-Manzanares, Luis Martínez-Dolz, Manuel Portolés, Esther Roselló-Lletí

**Affiliations:** 1 Clinical and Translational Research in Cardiology Unit, Health Research Institute, Hospital La Fe, 46026 Valencia, Spain; lorena_perezc@iislafe.es (L.P.-C.); igies@alumni.uv.es (I.G.-E.); maria.garcia8@uchceu.es (M.G.-M.); martinez_luidol@gva.es (L.M.-D.); portoles_man@gva.es (M.P.); 2 Biomedical Research Networking Center on Cardiovascular Diseases (CIBERCV), Institute of Health Carlos III, 28029 Madrid, Spain; 3 Department of Animal Medicine and Surgery, Veterinary Faculty, Cardenal Herrera-CEU University, 46115 Valencia, Spain; 4 Failure and Transplantation Unit, Cardiology Department, University and Polytechnic La Fe Hospital, 46026 Valencia, Spain

**Keywords:** ischaemic cardiomyopathy, DNMT3B, DNA methylation, non-coding RNA

## Abstract

A controversial understanding of the state of the DNA methylation machinery exists in ischaemic cardiomyopathy (ICM). Moreover, its relationship to other epigenetic alterations is incomplete. Therefore, we carried out an in-depth study of the DNA methylation process in human cardiac tissue. We showed a dysregulation of the DNA methylation machinery accordingly with the genome-wide hypomethylation that we observed: specifically, an overexpression of main genes involved in the elimination of methyl groups (*TET1*, *SMUG1),* and underexpression of molecules implicated in the maintenance of methylation (*MBD2*, *UHRF1)*. By contrast, we found *DNMT3B* upregulation, a key molecule in the addition of methyl residues in DNA, and an underexpression of miR-133a-3p, an inhibitor of *DNMT3B* transcription. However, we found many relevant alterations that would counteract the upregulation observed, such as the overexpression of *TRAF6*, responsible for Dnmt3b degradation. Furthermore, we showed that molecules regulating Dnmts activity were altered; specifically, SAM/SAH ratio reduction. All these results are in concordance with the Dnmts normal function that we show. Our analysis revealed genome-wide hypomethylation along with dysregulation in the mechanisms of addition, elimination and maintenance of methyl groups in the DNA of ICM. We describe relevant alterations in the DNMT3B system, which promote a normal Dnmt3b function despite its upregulation.

## 1. Introduction

Ischaemic cardiomyopathy (ICM) is an important cause of mortality worldwide, associated with the development of heart failure (HF) [1]. Therefore, there is a great interest in the study of ICM to obtain a better understanding of the pathophysiology underlying the disease. One of the main research foci is epigenomics, which has allowed the identification of epigenetic alterations in ICM patients [2,3,4]. These epigenetic changes are associated with differential expression at the mRNA level of genes described in the development of ICM [5,6,7]. In addition, it has been observed that the epigenetic alterations produced during disease development could be valuable diagnostic and prognostic markers, and serve as therapeutic targets [8,9,10,11]. 

The most studied epigenetic modification is DNA methylation. It is a complex process, characterised by two main mechanisms: the addition and the elimination of methyl residues in DNA. The addition of methyl groups to the cytosine residues of DNA, which is associated with gene repression, is directly catalysed by three members of the DNA methyltransferase (DNMT) family. These molecules require S-adenosylmethionine (SAM), a methyl group donor, to carry out the reaction, which is then transformed into S-adenosyl-L-homocysteine (SAH). On the other hand, the removal of methyl groups from DNA is a complex process since several enzymatic reactions are required [12]. Moreover, in recent years, a large number of molecules implicated in DNA methylation have been described [13]. These key molecules can be classified based on their main function, as molecules involved in the addition, elimination and maintenance of the methyl residues on DNA or regulators of the methylation process. Other epigenetic mechanisms, such as post-translational modifications of histones and RNA-based mechanisms, mainly miRNAs, can also interact with the DNA methylation machinery. This allows complex gene regulation through the different components of the epigenome [14,15].

In the context of HF, an increase in the expression of the de novo *DNMTs*, *DNMT3A* and *DNMT3B*, has been observed [16], *DNMT3B* being the main DNA methyltransferase expressed in human and mouse hearts [17]. Controversially, greater hypomethylation has been observed in regions associated with genes that encode proteins in these patients [18]. Moreover, blood genome-wide DNA hypomethylation has been described to be a risk factor for ischaemic heart disease [19]. 

Therefore, due to the lack of knowledge and the controversies described related to the DNA methylation process in ICM, this study aims to elucidate the state of the DNA methylation machinery. To carry it out, we analyzed the expression of the main genes involved in DNA methylation in ischaemic hearts. Furthermore, to clarify the role of *DNMT3B* upregulation, we studied the state of the DNMT3B system and the genome-wide methylation state.

## 2. Materials and Methods

### 2.1. Cardiac Tissue Samples

Left ventricular (LV) tissue samples from 40 explanted human hearts were used in our experiments, some of them matching between different analyses. The specific sample size of each individual study is reported in Table 1. Patients were diagnosed with ICM based on the following inclusion criteria: prior documented episodes of acute myocardial infarction, an echocardiography showing normal contractility segments coexisting with other dyskinetic or akinetic segments and an electrocardiography showing signs of ischemia or myocardial necrosis. There were no signs of existence of a primary valvular disease. The ICM characteristics for inclusion in the study were obtained from clinical history, hemodynamic study, electrocardiograms and Doppler echocardiography data. All the data was collected by physicians who were blind to the subsequent analysis of the LV function. Patients were functionally classified following the New York Heart Association criteria and were receiving medical treatment in accordance with the guidelines of the European Society of Cardiology [20].

All controls (CNTs) had a normal LV function (LV ejection fraction >50%) and no history of any cardiac disease. Samples were obtained from non-diseased donor hearts that had been rejected for cardiac transplantation due to size or blood type incompatibility. All heart donors died either from a cerebrovascular accident or in a motor vehicle accident. 

Tissue samples were collected from near the apex of the left ventricle, maintained in 0.9% NaCl, and preserved at 4 °C for a maximum of 4.4 ± 3 h after coronary circulation loss. The samples were stored at −80 °C until further use. Appropriate handling and rapid sample collection and storage led to high-quality samples (DNA ratios 260/280 ~1.8 and 260/230 ~2.0, RNA ratio 260/280 ~2.0 and RNA integrity number ≥9).

This study was approved by the Ethics Committee (Biomedical Investigation Ethics Committee of La Fe University Hospital of Valencia, Spain) and was conducted in accordance with the guidelines of the Declaration of Helsinki [21]. Signed informed consent was obtained from each patient or, in the case of CNT subjects, from their relatives.

### 2.2. mRNA Extraction and Sequencing

For this analysis, 23 samples were used (ICM, *n* = 13; and CNT, *n* = 10). RNA isolation and RNA-sequencing (RNA-seq) procedures and analyses have been extensively described previously by Roselló-Lletí et al. [22]. Briefly, RNA extractions were performed using a PureLink™ Kit (Ambion Life Technologies; Waltham, MA, USA) and cDNA libraries were obtained following Illumina’s recommendations. Transcriptome libraries were sequenced on the SOLiD 5500 XL (Applied Biosystems; Waltham, MA, USA) platform. All data presented in this manuscript has been deposited in the NCBI’s Gene Expression Omnibus (GEO) database and are accessible through the GEO series accession number GSE55296.

### 2.3. ncRNA Extraction and Sequencing

For this analysis, 30 samples were used (ICM, *n* = 22; and CNT, *n* = 8). RNA extraction was carried out using the Quik-RNATM miniprep plus kit (Zymo Research; Irvine, CA, USA) and following the manufacturer’s instructions. RNA quantification was performed using NanoDrop 1000 spectrophotometer and Qubit 3.0 fluorometer (Thermo Fisher Scientific; Horsham, UK). The purity and the integrity of the RNA samples were determined using a 0.8% agarose gel and the Agilent 2100 Bioanalyzer with RNA 6000 nano assay and small RNA assay kits (Agilent Technologies; Spain). 

The cDNA libraries have been obtained following Illumina´s recommendations. Briefly, 3′ and 5′ adaptors were sequentially ligated to the RNA prior to reverse transcription and cDNA generation. The cDNA was enriched using PCR to create an indexed double-stranded cDNA library, and size selection was performed using a 6% polyacrylamide gel. The quality and quantity of the libraries were analyzed using a 4200 TapeStation (Agilent Technologies; Madrid, Spain) D1000 High-Sensitivity assay. The cDNA libraries were pooled and then sequenced using paired-end sequencing (100 × 2) in the Illumina HiSeq 2500 (Illumina; San Diego, CA, USA) sequencer.

### 2.4. Validation for RT-qPCR

Real-time quantitative polymerase chain reaction (RT-qPCR) was performed to validate the expression of *DNMT3B* in the LV samples (ICM, *n* = 14; and CNT, *n* = 8). One microgram of total RNA was reverse transcribed into cDNA using the M-MLV enzyme, as per the manufacturer’s instructions (Invitrogen; Waltham, MA, USA). The resulting cDNA was used as a template for RT-qPCR, which was performed in duplicate using the high-performance ViiATM 7 Real-Time PCR System thermal cycler, according to the manufacturer’s instructions (Applied Biosystems; Waltham, MA, USA) and using TaqMan^®^ (Thermo Fisher Scientific; Horsham, UK) probes: *DNMT3B* (Hs00171876_m1) and *GAPDH* (Hs99999905_m1). *GAPDH* was used as the reference gene. The relative expression of the DNMT3B gene was calculated according to the Livak method of 2^−ΔΔCt^ [23].

### 2.5. Enzyme-Linked Immunosorbent Assay

Dnmt3b protein levels, Dnmts activity and the concentrations of S-adenosylmethionine (SAM) and S-adenosylhomocysteine (SAH) were determined via a specific sandwich enzyme-linked immunosorbent assay, as per the manufacturer´s instructions (DNMT3B Assay Kit ab113471 from Abcam (Cambridge, UK), DNMT Activity Quantification Kit (Colorimetric) ab113467 from Abcam, Human S-adenosylmethionine SAM ELISA kit MBS2605308 and Human S-adenosylhomocysteine SAH ELISA kit MBS2602645 from MyBioSource (San Diego, CA, USA)). The tests were quantified at 450 nm in a dual-wavelength microplate reader (Sunrise; Tecan, Tecan Ibérica Instrumentación S.L Barcelona, Spain) using Magellan version 2.5 software (Tecan). 

Dnmt3b protein levels and Dnmts activity were measured in nuclear extracts from homogenate heart tissue (ICM, *n* = 8; CNT, *n* = 8). The isolation method of the nuclear fraction is detailed by Tarazón et al. [24]. Briefly, 100–150 mg of frozen left ventricle was used for the extraction, which was homogenized in an extraction buffer. The homogenates were subjected to a sucrose gradient and to successive ultracentrifugations until the isolation of the nuclear fraction. The concentrations of SAM and SAH were measured in cardiac tissue homogenates (ICM, *n* = 30; and CNT, *n* = 6). For the extraction, we used 25 mg of the frozen left ventricle. These were then homogenized with an extraction buffer in a FastPrep-24 homogenizer (MP Biomedicals; Irvine, CA, USA) with specifically designed Lysing Matrix D tubes. The homogenates were centrifuged, and the supernatant was aliquoted. The SAM and SAH tests have a limit of detection up to 5 ng/mL and 0.06 ng/mL, respectively. No significant cross-reactivity or interference between these metabolites and their analogues were observed.

### 2.6. DNA Extraction and Infinium MethylationEPIC BeadChip

For this analysis, 16 samples were used (ICM, *n* = 8; and CNT, *n* = 8). DNA isolation and DNA methylation analyses—using the Infinium MethylationEPIC BeadChip platform (Illumina; San Diego, CA, USA) to interrogate over 850,000 CpG sites—were performed and extensively described by Ortega et al. [7]. 

### 2.7. Statistical Methods

Data are expressed as the mean ± standard deviation (SD) of the mean for continuous variables and as percentage values for discrete variables. The Kolmogorov–Smirnov test was used to analyze the data distribution. Significant differences between the means of groups with a normal distribution were analyzed using the Student’s *t*-test, while the non-parametric Mann–Whitney U test was used for data that was non-normally distributed. Finally, Pearson’s correlation coefficient was calculated to determine the statistical relationship between variables. The molecules with *p* < 0.05 and fold > 1.20 were considered statistically significant. CpGs with a Δβ ≥ ±0.1 and *p* < 0.05 were considered as differentially methylated. All statistical analyses were performed using the SPSS software (version 20.0) for Windows (IBM SPSS Inc.; Endicott, NY, USA).

## 3. Results

### 3.1. Clinical Characteristics of Patients

This study included explanted heart tissue samples from CNT individuals and HF patients from ischaemic etiology. The six study populations with ICM were homogeneous with regard to their clinical characteristics (Table 1). ICM patients had a mean age of 56 ± 8 years, and 99% were male. They belonged to classes III–IV of the New York Heart Association functional classification and presented altered values in echocardiographic parameters. Furthermore, they were previously diagnosed with significant comorbidities, including hypertension and diabetes mellitus. CNT individuals had a mean age of 55 ± 17 years, and 65% were male. Comorbidities and other echocardiographic data were not available for the CNT group, in accordance with the Spanish Organic Law on Data Protection 15/1999. 

### 3.2. mRNA Expressio£n of Genes Involved in DNA Methylation

**Figure 1:**
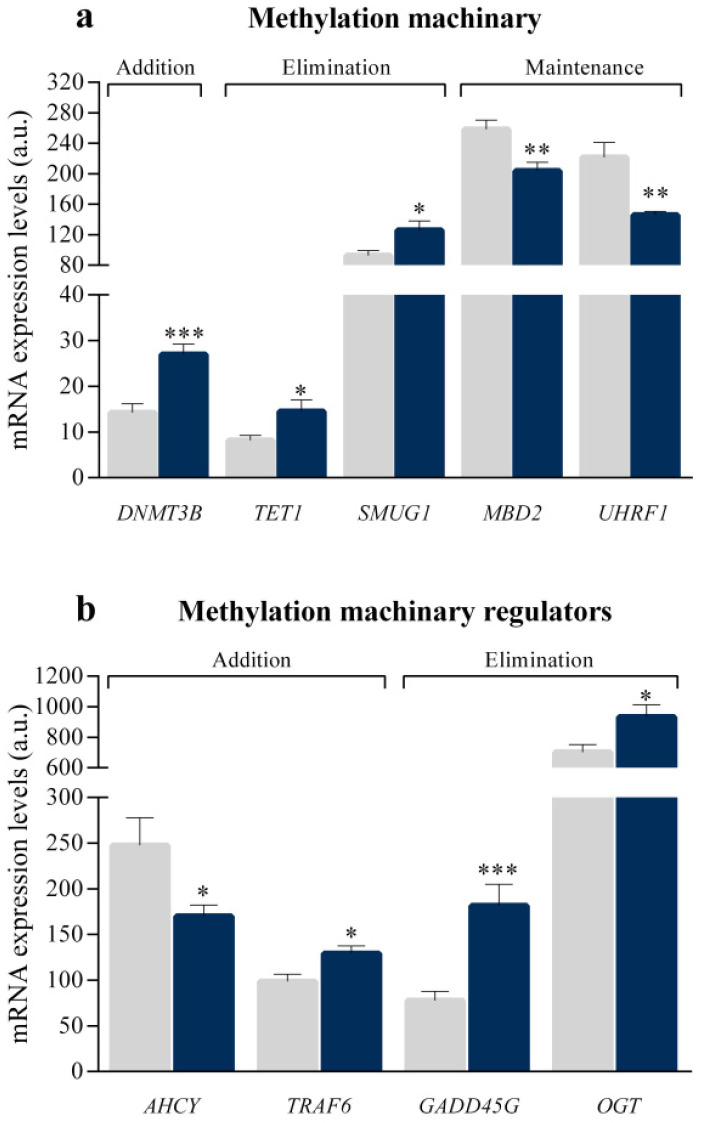
mRNA relative expression levels of altered genes involved in the DNA methylation process in ischaemic hearts versus controls. (**a**) Methylation machinery of DNA: genes related to addition (*DNMT3B*), elimination (*TET1* and *SMUG1*) and maintenance (*MBD2* and *UHRF1*) of methyl groups to DNA. (**b**) Methylation machinery regulators (*AHCY*, *TRAF6*, *GADD45G* and *OGT*). The results were obtained by mRNA-sequencing SOLiD 5500XL platform. Data are presented as the mean ± SEM. a.u., arbitrary units. Ischaemic cardiomyopathy patients (*n* = 13; blue), controls subjects (*n* = 10; grey). * *p* < 0.05, ** *p* < 0.01, *** *p* < 0.001.

Transcriptome-level differences between ICM and CNT samples were investigated using the mRNA-seq technology. We focused on the study of the genes involved in the DNA methylation machinery; these genes were classified based on their main function, relating to the addition, elimination, maintenance of methyl residues in DNA, or such as regulators of the DNA methylation process (Supplementary Table S1). We found alterations in several molecules of the methylation machinery (Figure 1a). Specifically, we observed the overexpression in *DNMT3B* gene (1.90 fold and *p* < 0.001), a molecule involved in the addition of de novo methyl groups in the DNA. Amongst the major genes analyzed associated with the elimination of methyl groups that were expressed in the heart, we observed that *TET1* (1.94 fold and *p* < 0.05) and *SMUG1* (1.36 fold and *p* < 0.05) were overexpressed in ICM patients. In addition, *MBD2* (−1.27 fold and *p* < 0.01) and *UHRF1* (−1.52 fold and *p* < 0.01) genes were underexpressed, both implicated in the maintenance of methyl groups. Finally, genes involved in the regulation of the methylation process were analyzed (Figure 1b). Specifically, we observed alterations in genes implicated in the regulation of the addition of methyl group, *AHCY* (−1.46 fold and *p* < 0.05) and *TRAF6* (1.31 fold and *p* < 0.05), and overexpression of genes related to the regulation of the elimination of methyl groups, *GADD45G* (2.33 fold and *p* < 0.001) and *OGT* (1.33 fold and *p* < 0.05).

### 3.3. DNMT3B Regulation Analysis 

**Figure 2:**
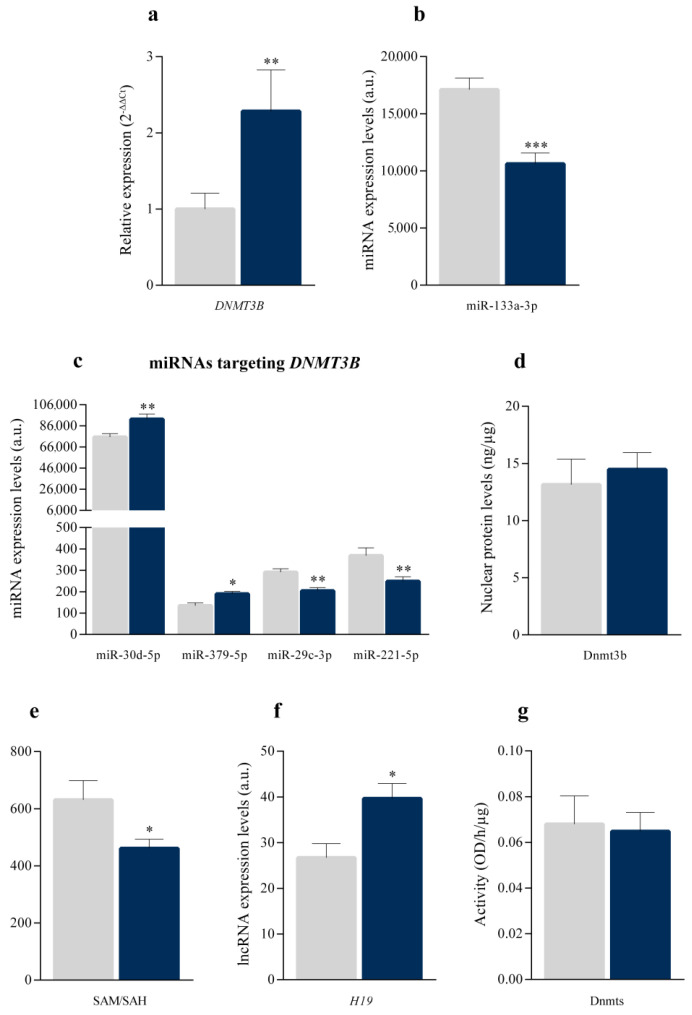
DNMT3B system dysregulation. (**a**) *DNMT3B* mRNA levels in ischaemic hearts (*n* = 14) and controls (*n* = 8), using RT-qPCR. (**b**) miR-133a-3p expression levels in ischaemic hearts (*n* = 22) and controls (*n* = 8), using HiSeq 2500 platform. (**c**) miRNAs expression levels in ischaemic hearts (*n* = 22) and controls (n = 8), using HiSeq 2500 platform. (**d**) Dnmt3b nuclear protein levels in ischaemic hearts (*n* = 8) and controls (*n* = 8), using ELISA. (**e**) SAM/SAH ratio in ischaemic hearts (*n* = 30) and controls (*n* = 6), using ELISA. (**f**) lncRNA *H19* expression level in ischaemic hearts (*n* = 22) and controls (*n* = 8), using the Illumina HiSeq 2500 platform. (**g**) Dnmts nuclear activity in ischaemic hearts (*n* = 8) and controls (*n* = 8), using ELISA. Data are presented as the mean ± SEM. a.u., arbitrary units. Ischaemic cardiomyopathy patients (blue), controls subjects (grey). * *p* < 0.05, ** *p* < 0.01, *** *p* < 0.001.

We validated the expression of *DNMT3B*, which plays a central role in the DNA methylation process and its overexpression contrasted with the general hypomethylation observed in these ICM patients. The results obtained by RT-qPCR corroborated the *DNMT3B* overexpression (2.22 fold and *p* < 0.01; Figure 2a) observed by RNA-seq (Figure 1a). We carried out a second RNA sequencing study, which allows the detection of ncRNAs and we observed alterations in several miRNAs previously described as regulators of *DNMT3B* expression. miR-133a-3p (Figure 2b), negative regulator of *DNMT3B* transcription, was reduced (−1.38 fold and *p* < 0.001). In addition, post-transcriptional regulators miRNAs of *DNMT3B* were altered (Figure 2c). Specifically, the miR-30d-5p (1.22 fold and *p* < 0.01) and miR-379-5p (1.41 fold and *p* < 0.05) were overexpressed, while the miR-29c-3p (−1.30 fold and *p* < 0.01) and miR-221-5p (−1.33 fold and *p* < 0.01) were underexpressed in ICM patients. Next, nuclear levels of the Dnmt3b protein were analyzed and we did not observe statistically significant changes between ICM and control individuals (Figure 2d). 

On the other hand, we analyzed different molecules that regulate the activity of Dnmts to determine whether their levels could favor the inhibition of Dnmts and, consequently, the global hypomethylation in ICM patients. Lower levels of SAM and higher levels of SAH are related to reduce methylation capacity, which is represented by the SAM/SAH ratio [25]. We measured SAM and SAH levels in human heart samples through ELISA analyses. The SAM/SAH ratio (Figure 2e) was reduced by 37% in ICM patients (*p* < 0.05). Specifically, we obtained a SAM/SAH ratio of 631.45 ± 163.26 in the CNT group and 461.55 ± 173.72 in ICM patients. Moreover, as previously shown, *AHCY* was underexpressed in ICM patients (Figure 1b). This gene encodes the S-adenosyl-L-homocysteine hydrolase (SAHH) protein, which is responsible for degrading SAH. Furthermore, through the sequencing of ncRNAs, we detected the levels of lncRNA *H19* (Figure 2f), which was overexpressed in ICM patients (1.68 fold and *p* < 0.01). *H19* is an SAHH inhibitor that favors the accumulation of SAH in the cells. In addition, the expression of *H19* correlated with the ejection fraction (r = 0.590 and *p* < 0.01). Next, we analysed Dnmts activity, observing absence of changes in ICM patients compared to control individuals (Figure 2g).

### 3.4. State of Global DNA Methylation

**Figure 3:**
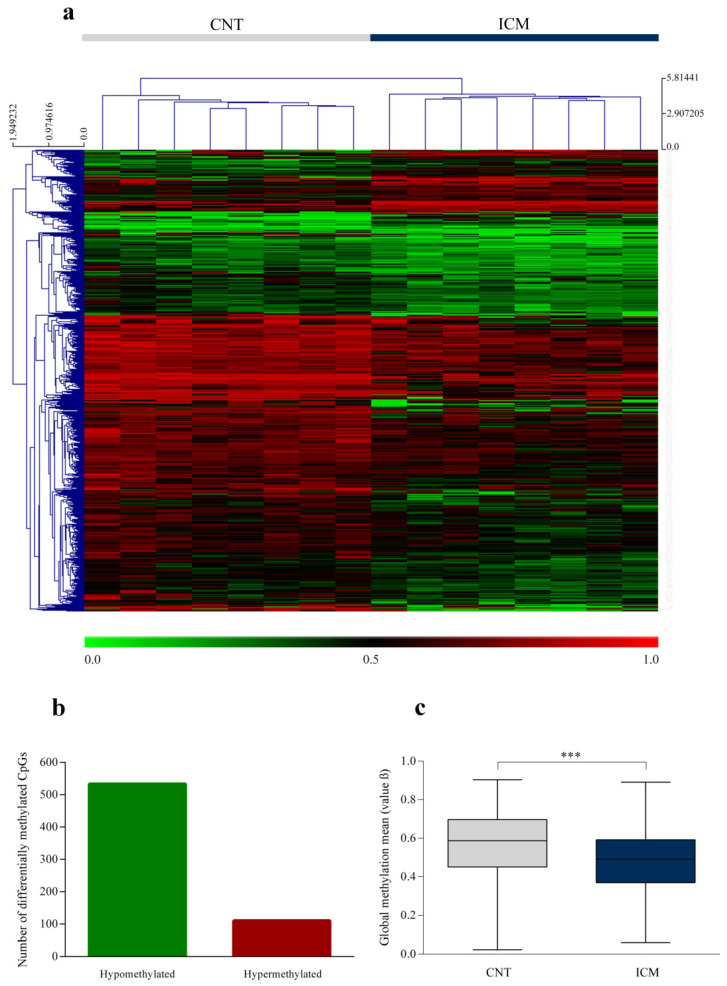
Global CpGs hypomethylation in ischaemic cardiomyopathy patients. (**a**) Heat map representing global methylation in ischaemic hearts (*n* = 8) and controls (*n* = 8) for each statistically significant CpGs. The results are represented as individual β values (1 = hypermethylated (red) and 0 = hypomethylated (green)). (**b**) Bar graph illustrating the number of hypermethylated and hypomethylated CpGs in ischaemic hearts. (**c**) Box plot graph with global methylation mean. Data are presented as the mean ± SEM. *** *p* < 0.0001. The results were obtained by the 850 K Infinium MethylationEPIC BeadChip platform.

We analyzed global DNA methylation levels in ICM patients and CNT individuals. The β value and the *p* value were calculated for each detected CpG site, allowing the identification of 643 differentially methylated CpGs sites. We represented each CpG with a statistically significant value using a heat map (Figure 3a), which identified the ICM and CNT groups in two different methylation patterns. Specifically, the 82.9% of the CpGs sites were hypomethylated in ICM patients (Figure 3b). Furthermore, we analyzed the global methylation mean between both groups (ICM, mean β value = 0.481 ± 0.164 and CNT, mean β value = 0.569 ± 0.171; *p* < 0.0001 (Figure 3c)). These results showed genome-wide hypomethylation in ICM patients. 

Next, we analyzed the position in the genome of the differentially methylated CpGs (Table 2). The 26.1% sites were distributed in the promoter proximal regions (TSS1500, TSS200 and 5´UTR), 44.3% in gene body and first exons, and 29.6% in 3´UTR and intergenic regions. In relation to CpG content and neighborhood context, the 54% of CpGs were present in open sea; the 27.2% in shores and shelves of CpG sites; and the 18.8% in CpGs sites. Although there is an evident greater number of a hypomethylated CpGs sites in ICM patients, we observed that the percentage of methylation is similar for hypermethylated and hypomethylated CpGs sites in each region, except for absence of differentially hypermethylated CpGs sites in 3´UTR regions and the marked reduction in the percentage of hypermethylation in the context of the TSS200 regions and islands.

## 4. Discussion

Gene expression is regulated by pathological mechanisms involving DNA methylation and ncRNAs function. Both processes are closely related, but incomplete knowledge of these epigenetic alterations exists in ICM [26]. Moreover, controversial results have been published regarding DNA methylation in ICM. Specifically, increased expression of methyl group addition molecules contrasts with the DNA hypomethylation observed [16,18]. Thus, this study focused on the analysis of the state of the DNA methylation machinery in these patients. Our results showed an alteration of the DNA methylation machinery in ICM patients; specifically, we found a dysregulation in addition, elimination and maintenance of methyl groups accordingly with the genome-wide hypomethylation observed. 

Regarding the mechanisms of elimination of methyl groups, we observed the overexpression of *TET1* and *SMUG1* genes, both molecules favoring DNA hypomethylation [27]. *TET1* plays a major role in the initiation of the active DNA demethylation process [28,29]. Furthermore, *OGT* has been related to the post-transcriptional regulation of *TET1* in animal models [30]. Loss of Ogt in early fetal cardiomyocytes leads to multiple heart developmental defects, including hypertrabeculation, biventricular dilation, atrial septal defects, ventricular septal defects and defects in coronary vessel development [31]. In this study, we observed overexpression of the *OGT* gene. Ogt adds residues of O-GlcNAc to Tet1, thereby stimulating its activity [32]. However, the impact of Ogt on the Tet family in human cells is slightly controversial and requires additional studies [33]. In addition, we observed overexpression of the *GADD45G* gene in ICM. This gene belongs to the *GADD45* gene family, which participates in the regulation of the active demethylation process, and its expression is stimulated by cellular stress [34]. Moreover, Lucas et al. [35] demonstrated that Gadd45g is up-regulated in murine cardiomyocytes subjected to simulated ischemia, and that it promotes the development and persistence of HF by inducing cardiomyocyte apoptosis. These results indicate a dysregulation in the process of elimination of methyl groups, which could favor the demethylation of DNA. 

Furthermore, the *MBD2* and *UHRF1* genes were underexpressed in this study. These genes are responsible for reading the DNA methylation pattern and maintaining the methylated state associated with transcriptional repression [12]. Genetic alterations in *MBD2* and *UHRF1* have been linked to various cardiovascular pathologies such as atherosclerosis or arterial aneurism, which can trigger HF [36,37].

On the other hand, we observed overexpression of the *DNMT3B* gene in ICM patients. This gene encodes a methyltransferase responsible for adding methyl groups to DNA, thus promoting the methylated state. Previously, *DNMT3B* overexpression has been reported in the context of HF [16], which could be favored by hypoxic conditions [38]. *DNMT3B* is a molecule subject to significant regulation. At the transcriptional level, different miRNAs regulators of its expression have been described in human and mouse cells [39,40,41,42]. Specifically, the miR-133a-3p binds to the *DNMT3B* promoter, reducing its transcription in cardiac cells [43], finding in our results the underexpression of miR-133a-3p in ICM patients. Furthermore, we observed alterations in several *DNMT3B* target miRNAs, which can induce dysregulation in the levels of mRNA, as well as at the protein level. Next, we analysed Dnmt3b nuclear protein levels. Our results did not show differences at the protein level between patients with ICM and control individuals. Yu et al. [44] have described in murine models of breast cancer that the ubiquitin E3 ligase Traf6 interacts with Dnmts, catalyzing their ubiquitination and inducing protein degradation. *TRAF6* mRNA levels were increased in ICM patients, being able to favor lysosomal degradation of Dnmt3b.

Moreover, the enzymatic activity of Dnmt3b is regulated by different molecules. Specifically, the SAH metabolite acts as an inhibitor of various methyltransferases, including Dnmt3b, thereby preventing the addition of methyl groups to DNA [45,46]. The accumulation of SAH in cells is caused by the SAM-dependent methylation, whose degradation product is SAH, and by the inactivation of SAHH, a molecule involved in the breakdown of SAH into adenosine and homocysteine. The results obtained in this study showed that the SAM/SAH ratio was reduced in ICM patients. SAH is a competitive inhibitor of Dnmts; therefore, the increase in SAH, together with the decrease in SAM, favors the binding of this molecule to one of its main targets. In addition, the reduction in SAM/SAH ratio has been linked to the development of vascular diseases and to DNA hypomethylation of the genome [47]. These results reinforce the existence of a generalized DNA hypomethylation in ICM patients. Furthermore, we observed the underexpression of *AHCY*, gene encoding SAHH and the overexpression of lncRNA *H19* in ICM patients. *H19* acts as an SAHH inhibitor, thus preventing SAH degradation [48]. However, the Dnmts activity was similar in ICM patients and control individuals. In recent years, the role of *H19* in the development and prognosis of HF has been extensively studied [49,50]. Several authors described altered *H19* expression in cardiovascular diseases and related it with hypertrophic growth inhibition in cardiomyocytes and alleviation of myocardial ischemia reperfusion injury [51,52,53]. We further observed that its expression related with ejection fraction, the central measure of left ventricular function, as previously has been described [52]. These results represent a promising possibility to identify new therapeutic strategies, such as *H19*-targeted delivery for clinical ICM therapy.

To date, alterations in the methylation pattern have been identified in ICM patients in specific regions of the genome [16]. Moreover, the analysis of genome-wide DNA methylation in human ICM was carried out, observing differential methylation in the CpG sites associated with gene promoters [54], as well as a greater hypomethylation in regions associated with genes that encode proteins in these patients [18]. We also analyzed the status of the CpG sites as a whole in ICM patients compared to control individuals, and our results showed a higher degree of hypomethylated CpGs in each of the genomic regions. In addition, the results obtained in the present study indicate an increase in the regulatory mechanisms that promote DNA demethylation. Consistently, our assessment of the global DNA methylation pattern revealed that explanted hearts from individuals with ICM presented genome-wide hypomethylation. A limitation of this study includes the use of cardiac tissue samples from patients with end-stage HF, who exhibit intrinsic variability that exists between individuals. The patients who participated in this study were also treated pharmacologically, and some therapies may have influenced the results. However, our study population was etiologically homogeneous and all individuals had been receiving medical treatment according to the guidelines of the European Society of Cardiology [20]. 

## 5. Conclusions

Our analysis revealed genome-wide hypomethylation concordant with dysregulation in the mechanisms of addition, elimination and maintenance of methyl groups in DNA observed in ischaemic hearts. We describe relevant alterations in the DNMT3B system, which promote a normal Dnmt3b function despite its upregulation.

## Figures and Tables

**Table 1:** Clinical characteristics of ischaemic cardiomyopathy patients.

	Epigenomic Study	mRNA Sequencing	ncRNA Sequencing	*DNMT3B* Validation	Dnmt3b Nuclear Protein *	SAM/SAH Ratio
	*n* = 8	*n* = 13	*n* = 22	*n* = 14	*n* = 8	*n* = 30
Age (years)	53 ± 5	54 ± 8	55 ± 8	55 ± 8	53 ± 6	55 ± 8
Gender male (%)	100	100	100	93	100	100
*NYHA* class	III–IV	III–IV	III–IV	III–IV	III–IV	III–IV
BMI (kg/m^2^)	28 ± 3	27 ± 4	26 ± 3	27 ± 4	28 ± 4	27 ± 4
Haemoglobin (mg/dL)	14 ± 2	14 ± 3	14 ± 2	13 ± 3	15 ± 2	14 ± 2
Haematocrit (%)	44 ± 4	41 ± 6	41 ± 6	40 ± 8	43 ± 4	41 ± 5
Total cholesterol (mg/dL)	152 ± 43	162 ± 41	174 ± 45	160 ± 40	162 ± 46	187 ± 44
Prior hypertension (%)	25	33	40	31	38	56
Prior smoking (%)	88	92	81	85	88	77
Diabetes mellitus (%)	63	42	45	38	50	55
LVEF (%)	24 ± 6	25 ± 5	24 ± 7	24 ± 6	23 ± 5	24 ± 7
LVESD (mm)	57 ± 8	57 ± 8	54 ± 8	57 ± 8	57 ± 8	54 ± 8
LVEDD (mm)	65 ± 7	65 ± 8	63 ± 9	65 ± 8	65 ± 8	63 ± 8

BMI: body mass index; ICM: ischaemic cardiomyopathy; LVEF: left ventricular ejection fraction; LVEDD: left ventricular end-diastolic diameter; LVESD: left ventricular end-systolic diameter; NYHA: New York Heart Association. Data are presented as the mean ± SD.* The same patients were used to measure Dnmts nuclear activity.

**Table 2:** Comparison of statistically significant CpG site methylation (Δβ > 0.1 and *p* < 0.05) between ischaemic cardiomyopathy patients and control individuals.

**Functional Genomic Distribution**						
	**All CpG Sites**		**Hypermethylated** **CpG Sites**		**Hypomethylated** **CpG Sites**	
	CpGs	%	CpGs	%	CpGs	%
TSS1500	67	10.4	11	10.0	56	10.5
TSS200	47	7.3	3	2.7	44	8.3
5’UTR	54	8.4	9	8.2	45	8.4
1stExon	26	4.0	5	4.5	21	3.9
Gene body	259	44.3	49	44.5	210	39.4
3’UTR	16	2.5	0	0.0	16	3.0
Intergenic	174	27.1	33	30.0	141	26.5
	643		110		533	
**CpG Content and Neighbourhood Context**						
	**All CpG Sites**		**Hypermethylated** **CpG Sites**		**Hypomethylated** **CpG Sites**	
	CpGs	%	CpGs	%	CpGs	%
North Shelf	28	4.3	8	7.3	20	3.8
Surth Shelf	19	3.0	4	3.6	15	2.8
North Shore	75	11.7	17	15.5	58	10.9
Surth Shore	53	8.2	8	7.3	45	8.4
Island	121	18.8	10	9.1	111	20.8
Open sea	347	54.0	63	57.3	284	53.3
	643		110		533	

## Data Availability

The mRNA-seq data discussed in this publication have been deposited in NCBI’s Gene Expression Omnibus and are accessible through GEO Series accession number GSE55296 (http://www.ncbi.nlm.nih.gov/geo/query/acc.cgi?acc=GSE55296, accessed on 11 March 2022).

## Supplementary Materials

The following supporting information can be downloaded at: https://www.mdpi.com/article/10.3390/biomedicines10040866/s1, Table S1: Genes involved in DNA methylation process in human heart.
