# Supplementary material for: DNMT3B System Dysregulation Contributes to the Hypomethylated State in Ischaemic Human Hearts
Source: Biomedicines. 2022 Apr 7;10(4):866. doi: 10.3390/biomedicines10040866 (PMC9029641; doi:10.3390/biomedicines10040866)
Supplement: Supplementary file 1 [file biomedicines-10-00866-s001.zip › biomedicines-1654997-supplementary.pdf]

Table S1. Genes involved in DNA methylation process in human heart.

	Gene ID	Gene Name	Protein Name (UniProtKB)
**DNA methylation machinery**			
**Addition of methyl groups**			
1	ENSG00000130816	*DNMT1*	DNA (cytosine-5)-methyltransferase 1
2	ENSG00000119772	*DNMT3A*	DNA (cytosine-5)-methyltransferase 3A
3	ENSG00000088305	*DNMT3B*	DNA (cytosine-5)-methyltransferase 3B
**Elimination of methyl groups**			
4	ENSG00000239713	*APOBEC3G*	DNA dC->dU-editing enzyme APOBEC-3G
5	ENSG00000244509	*APOBEC3C*	DNA dC->dU-editing enzyme APOBEC-3C
6	ENSG00000128394	*APOBEC3F*	DNA dC->dU-editing enzyme APOBEC-3F
7	ENSG00000243811	*APOBEC3D*	DNA dC->dU-editing enzyme APOBEC-3D
8	ENSG00000123415	*SMUG1*	Single-strand selective monofunctional uracil DNA glycosylase
9	ENSG00000139372	*TDG*	G/T mismatch-specific thymine DNA glycosylase
10	ENSG00000138336	*TET1*	Methylcytosine dioxygenase TET1
11	ENSG00000168769	*TET2*	Methylcytosine dioxygenase TET2
12	ENSG00000187605	*TET3*	Methylcytosine dioxygenase TET3
**Maintenance of methyl groups**			
13	ENSG00000141644	*MBD1*	Methyl-CpG-binding domain protein 1
14	ENSG00000134046	*MBD2*	Methyl-CpG-binding domain protein 2
15	ENSG00000071655	*MBD3*	Methyl-CpG-binding domain protein 3
16	ENSG00000129071	*MBD4*	Methyl-CpG-binding domain protein 4
17	ENSG00000169057	*MECP2*	Methyl-CpG-binding protein 2
18	ENSG00000065060	*UHRF1*	E3 ubiquitin-protein ligase UHRF1
19	ENSG00000147854	*UHRF2*	E3 ubiquitin-protein ligase UHRF2
20	ENSG00000174282	*ZBTB4*	Zinc finger and BTB domain-containing protein 4
21	ENSG00000177485	*ZBTB33*	Transcriptional regulator Kaiso
22	ENSG00000177311	*ZBTB38*	Zinc finger and BTB domain-containing protein 38
**Regulation of methylation**			
**Regulation of the addition of methyl groups**			
23	ENSG00000101444	*AHCY*	Adenosylhomocysteinase
24	ENSG00000099381	*KMT2A*	Histone-lysine N-methyltransferase 2[a]
25	ENSG00000159200	*RCAN1*	Calcipressin-1
26	ENSG00000172348	*RCAN2*	Calcipressin-2
27	ENSG00000117602	*RCAN3*	Calcipressin-3
28	ENSG00000099381	*SETD1A*	Histone-lysine N-methyltransferase SETD1A
29	ENSG00000181555	*SETD2*	Histone-lysine N-methyltransferase SETD2
30	ENSG00000143379	*SETDB1*	Histone-lysine N-methyltransferase SETDB1
31	ENSG00000101945	*SUV39H1*	Histone-lysine N-methyltransferase SUV39H1
32	ENSG00000175104	*TRAF6*	TNF receptor-associated factor 6
**Regulation of the elimination of methyl groups**			
33	ENSG00000108468	*CBX1*	Chromobox protein homolog 1
34	ENSG00000154832	*CXXC1*	CXXC-type zinc finger protein 1

35	ENSG00000108799	*EZH1*	Histone-lysine N-methyltransferase EZH1
36	ENSG00000106462	*EZH2*	Histone-lysine N-methyltransferase EZH2
37	ENSG00000167491	*GATAD2A*	Transcriptional repressor p66-alpha
38	ENSG00000167491	*GATAD2B*	Transcriptional repressor p66-beta
39	ENSG00000116717	*GADD45A*	Growth arrest and DNA damage-inducible protein GADD45 alpha
40	ENSG00000099860	*GADD45B*	Growth arrest and DNA damage-inducible protein GADD45 beta
41	ENSG00000130222	*GADD45G*	Growth arrest and DNA damage-inducible protein GADD45 gamma
42	ENSG00000196712	*NF1*	Neurofibromin
43	ENSG00000186575	*NF2*	Merlin
44	ENSG00000147162	*OGT*	UDP-N-acetylglucosamine-peptide N-acetylglucosaminyltransferase 110 kDa subunit
45	ENSG00000169375	*SIN3A*	Paired amphipathic helix protein Sin3a
46	ENSG00000127511	*SIN3B*	Paired amphipathic helix protein Sin3b
47	ENSG00000078900	*TP73*	Tumor protein p73
**Regulation of the maintenance of methyl groups**			
48	ENSG00000145391	*SETD7*	Histone-lysine N-methyltransferase SETD7
49	ENSG00000183955	*SETD8*	Histone-lysine N-methyltransferase SETD8

APOBEC, apolipoprotein B mRNA-editing catalytic polypeptide-like; TET, Ten-eleven translocation; UHRF, ubiquitin like with PHD and ring finger domains 1; BTB, Broad-Complex, Tramtrack and Bric a brac; SUV39H1, Suppressor of variegation 3-9 Homolog 1; SETD1A, Su(var)3-9, Enhancer-of-zeste, Trithorax domain containing 1A; SETDB1, Su(var)3-9, Enhancer-of-zeste, Trithorax domain bifurcate 1; CXXC, Cys-X-X-Cys; SETD2; Su(var)3-9, Enhancer-of-zeste, Trithorax domain containing 2.
